# Network-level mechanisms underlying effects of transcranial direct current stimulation (tDCS) on visuomotor learning impairments in schizophrenia

**DOI:** 10.21203/rs.3.rs-2711867/v1

**Published:** 2023-04-06

**Authors:** Daniel Javitt, Pejman Sehatpour, Johanna Kreither, Javier Lopez-Calderon, Adithya Shastry, Heloise De-Baun, Antigona Martinez

**Affiliations:** Columbia University Medical Center/Nathan Kline Institute; Columbia University Medical Center/Nathan Kline Institute

## Abstract

Motor learning is a fundamental skill to our daily lives. Dysfunction in motor performance in schizophrenia (Sz) is associated with poor social and functional outcomes, but nevertheless remains understudied relative to other neurocognitive domains. Moreover, transcranial direct current stimulation (tDCS) can influence underlying brain function in Sz and may be especially useful in enhancing local cortical plasticity, but underlying neural mechanisms remain incompletely understood. Here, we evaluated performance of Sz individuals on the Serial Reaction Time Task (SRTT), which has been extensively used in prior tDCS research, in combination with concurrent tDCS and EEG source localization first to evaluate the integrity of visuomotor learning in Sz relative to other cognitive domains and second to investigate underlying neural mechanisms. Twenty-seven individuals with Sz and 21 healthy controls (HC) performed the SRTT task as they received sham or active tDCS and simultaneous EEG recording. Measures of motor, neuropsychological and global functioning were also assessed. Impaired SRTT performance correlated significantly with deficits in motor performance, working memory, and global functioning. Time-frequency (“Beamformer”) EEG source localization showed beta-band coherence across supplementary-motor, primary-motor and visual cortex regions, with reduced visuomotor coherence in Sz relative to HC. Cathodal tDCS targeting both visual and motor regions resulted in significant modulation in coherence particularly across the motor-visual nodes of the network accompanied by significant improvement in motor learning in both controls and patients. Overall, these findings demonstrate the utility of the SRTT to study mechanisms of visuomotor impairment in Sz and demonstrate significant tDCS effects on both learning and connectivity when applied over either visual or motor regions. The findings support continued study of dysfunctional dorsal-stream visual connectivity and motor plasticity as components of cognitive impairment in Sz, of local tDCS administration for enhancement of plasticity, and of source-space EEG-based biomarkers for evaluation of underlying neural mechanisms.

## Introduction

Schizophrenia (Sz) is a serious mental disorder and a leading cause of long-term disability. Impaired functional outcome is driven largely by impairments in cognitive function that persist despite treatment with best available medications (rev. in 1,2). In its initial formulation, the NIMH Research Domain Criteria (RDoC) Cognitive Systems domain focused especially on processes such as perception, cognitive control and working memory (3, 4), for which extensive cognitive tasks are presently available (e.g. 5). Subsequently, the need for a motor domain was recognized as well (6–9), although validated tests remain underdeveloped. In addition, non-invasive brain stimulation approaches such as transcranial magnetic stimulation (tDCS) are proposed as potential treatments for cognitive dysfunction especially by enhanced neuroplasticity (e.g. 10,11–13) although optimal approaches again need to be developed (e.g. 14,15–20). The Serial Reaction Time Task (SRTT) (also known as the serial finger tapping task, SFTT) has been widely used to study mechanisms of motor plasticity and tDCS effects across healthy and neurological populations (rev. in 21,22–24) but has been studied in Sz to only a limited degree (e.g. 25,26) and without associated biomarkers. Here, we evaluated the sensitivity of the SRTT task to neurocognitive dysfunction in Sz, as well as its sensitivity to tDCS and neurophysiological underpinnings in Sz individuals relative to healthy controls (HC).

**Figure 1:**
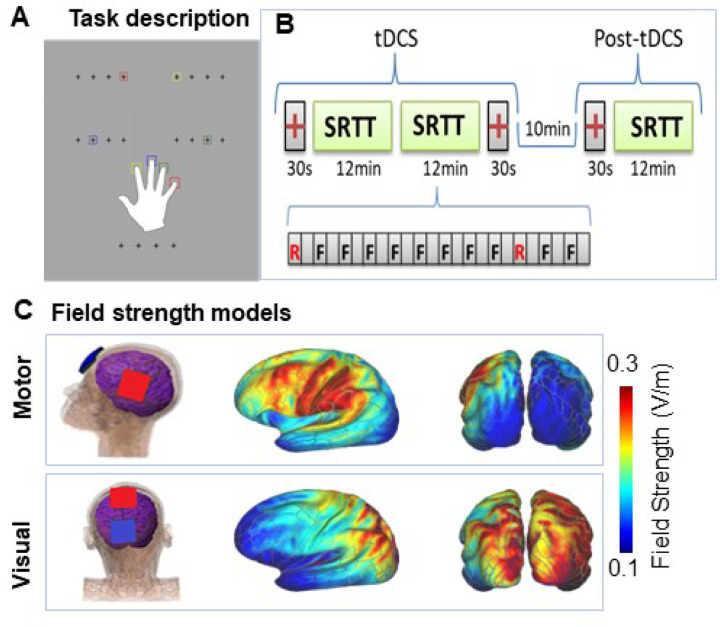
Paradigm and tDCS modeling. *A*. Schematic illustration of the task. Participants are instructed to react as quickly to colored squares in one of four positions denoted by crosses that remained on throughout the paradigm by pressing on a spatially and chromatically corresponding button on a keyboard. *B*. Task structure. The task used a 5-element repeat sequence that was modeled after previous studies (27,31,32). We used four different SRTT sequences (3, 1, 4, 2, 4), (2, 3, 1, 2, 4), (1, 3, 4, 2, 3), (4, 2, 1, 3, 2), pseudo randomly assigned to one of the four stimulation conditions i.e., Sham, Motor-anodal, Motor-cathodal and Visual-cathodal, per individual, such that no one received the same sequence twice. Two blocks of SRTT, 12 min each, were administered during tDCS/EEG. *C*. tDCS Field Strength Mapping. Pad placements for the Motor-cathodal and Motor-anodal conditions followed the M1-SO (left primary motor-right supraorbital) scalp positions used in prior tDCS SRTT studies. For Visual cortex stimulation, the anode pad was placed over the vertex (Cz) and the cathode pad was placed on the scalp area (POz) overlaying the cortical dorsal visual area (84). For sham stimulation, the pads were placed in the same positions as for motor stimulation; however, the stimulator only delivered 30 seconds of ramp up and down. The montage resulted in predominant current flow in premotor and somatomotor regions during motor cortex stimulation and dorsal visual and superior parietal regions during visual cortex stimulation (31).

In the SRTT, a fixed sequence of visual targets is presented repeatedly on a computer screen (Fig. 1A). When the sequence is random, the mean reaction time (RT) across trials remains relatively constant. In contrast, when the sequence repeats, individuals show a progressive reduction in RT over repeat trials even if they are not consciously aware of the sequence, reflecting implicit motor learning. The SRTT has been widely employed as an instrument to measure tDCS effects in part because of the ready accessibility of motor cortex to stimulation (rev. in 21). For example, tDCS stimulation over the primary motor cortex (M1) has been shown to increase learning when applied during the task (e.g. 27,28), whereas tDCS applied over parietal cortex enhances later stages of consolidation (29).

In the SRTT, the progressive reduction in RT during stimulus repetition primarily reflects a shift in individual responses from a slow, “reactive” mode (equivalent to a choice-reaction time task) in which the stimulus is needed to determine both where and when to press; to a fast “proactive” mode (equivalent to a simple reaction time task) in which the location of the stimulus has been predicted in advance and the stimulus indicates only when to press (30, 31). In HC, we have previously demonstrated that tDCS applied over either motor or visual cortex increases the shift from slow to fast responses along with changes in both EEG coherence and fMRI functional connectivity between visual and motor regions (31, 32). Here, we evaluate the degree to which similar effects can be achieved in Sz.

In Sz, cognitive impairments are assessed primarily using paper-and-pencil batteries such as the MATRICS consensus cognitive battery (MCCB) (33). While effective, such tasks are poorly suited to analyzing either the neural mechanisms underlying cognitive impairments or the potential mechanisms by which tDCS could reverse underlying dysfunction. An advantage of the SRTT is that the underlying cortical circuitry has been extensively evaluated and is known to depend upon the interaction of components of the motor cortex and the prefrontal supplementary motor area (SMA) region (12, 22, 34–38) with primary visual cortex (39) and the dorsal stream visual “action” system (40). Here, in order to interrelate SRTT performance to more traditional neurocognitive domains in Sz, we collected parallel data using both the MCCB and the Purdue Pegboard Test (41, 42), which serves as a test of both procedural learning and motor dysfunction across neuropsychiatric disorders.

At the electrophysiological level, interaction among regions involved in SRTT performance is indexed by coherent event-related desynchronization (ERD) of ongoing beta-frequency (10–24 Hz) rhythms within the extended motor network (e.g., 43,44–50). Task-dependent modulation of motor activity within the extended visuomotor networks, including in the SRTT, is reflected in alterations in coherence within the β (14–24 Hz) frequency range (35, 51, 52), as well as in fMRI functional connectivity between regions (31, 53). Nevertheless, optimal approaches for applying and guiding tDCS using neurophysiological brain measurements remain to be determined. To date, repeated tDCS targeted at specific brain regions has shown promise for treatment of specific symptomatic features, such as persistent auditory hallucinations (e.g. 54,55) or lack of insight (56). Nevertheless, studies seeking to use tDCS to enhance neuroplasticity in Sz have shown mixed success. For example, while some studies have found significant tDCS enhancement of LTP-like activity during repeated visual stimulation (57), others have reported negative results and have emphasized the need for further studies (58).

Against this background, the goal of the present study was three-fold. First, we evaluated the degree to which the SRTT may be useful in assessing neural mechanisms underlying specific aspects of motor dysfunction and neurocognitive dysfunction in Sz. Second, we evaluated the relative effectiveness of active vs. sham tDCS over motor and visual cortex in Sz relative to HC. Finally, we evaluated the degree to which β-coherence measures could be used to assess tDCS effects across populations.

Based upon prior findings of impaired dorsal stream visual function in Sz and its effects on higher level processing (59–65), we hypothesized that impaired SRTT performance in Sz would be related in part to impaired interaction of visual cortex with other nodes of the visuomotor system as well as local dysfunction within motor and premotor regions, and that beneficial effects would thus be obtained from tDCS applied over both motor and visual sensory regions. The present study builds on our prior combined neurophysiological/tDCS studies in HC (31, 32). To our knowledge this is the first study to evaluate either the neural underpinning of SRTT impairments or the neural mechanisms underlying pro-therapeutic effects of tDCS on neuroplasticity in Sz.

## Methods

### Participants

Participants included 21 healthy controls (HC) and 27 individuals with schizophrenia (Sz), aged 18–50 (Table 1). All participants provided written informed consent, and the procedures were approved by the Nathan Kline Institute/Rockland Psychiatric Center Institutional Review Board and ethics committee. All participants reported normal or corrected-to-normal vision. All were right-handed. Symptom ratings were obtained using the Positive and Negative Symptom Scale (PANSS). Neuropsychological assessment included the MATRICS consensus cognitive battery (MCCB) neurocognitive domains, and the Purdue Pegboard task(66, 67). Global function was assessed using the Independent Living Scale (ILS) (68) and Generalized assessment of function (GAF). Data from 3 of the HC were included in a prior report (31).

### Stimuli and Experimental Design

As previously described (31), stimuli consisted of colored squares that appeared in one of four positions, designated by crosses that collectively subtended ± 1.4 visual angle from the center of the screen. On each trial, participants pressed one of four visually cued color-coded keys on a standard computer keyboard with the fingers of their right hand as quickly and accurately as possible following presentation of a cue (Fig. 1A). Each block consisted of 12 self-paced 3-min runs, with random runs at positions 1 and 10 of the sequence (e.g. (27)). A single block was repeated 10-min post-tDCS (Fig. 1B).

### Behavioral data analysis

**Figure 2:**
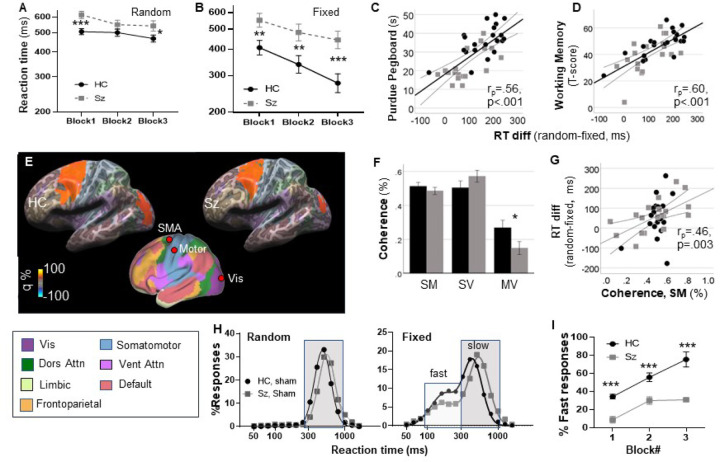
Between group comparison during sham stimulation. *A,B*. Reaction time (RT) by block for the random (*A*) and fixed (*B*) conditions. *C,D*. Correlations with indicated neuropsychological tests. *E*. Mean source space solutions for premotor β-event-related desynchronization (ERD) responses, showing the location of the supplemental motor area (SMA), motor, and visual (Vis) sources. q values represent normalized power within the time-frequency bin of interest. *F*. Coherence values for SMA-motor (SM), SMA-visual (SV) and motor-visual (MV) sources. *G*.Correlation between basal coherence in the SM connection and RT differences across groups. *H*. Single-trial RT histograms for HC and Sz participants in the random and fixed condition, pooled across blocks for calculation of mean RT values. *I*. Percent fast responses in the fixed condition as a function of Group and Block. *p<.05, **p<.01, ***p<.001.

For baseline analyses, RT data from random and fixed runs were log-transformed and averaged across trials within a block (Fig. 2). Mean values were compared across groups using repeated measures ANOVA with within-individual factor of Block and between-individual factor of Group status. Partial correlations controlling for group status were used to assess the relationship between RT and clinical data across participants. To assess effects of tDCS, a mixed-model regression was performed across runs, with run as a co-variate and Group membership and tDCS condition as factors.

For single-trial analyses, single-trial log-RT distributions were compared across conditions using single vs. dual-Gaussian models using GraphPad 9.0 non-linear curve fitting functions as described previously (31). For each analysis, both single Guassian and dual Gaussian fits were considered. For the single Guassian model, data were fit to the formula (# of responses@log-RT = X) = (Total # of responses across RTs) * e^−^ (X–Mean/SD^2), where Total number of responses, Mean, and SD were modeled parameters. All parameters were constrained to be positive values. For the dual Gaussian model, a second set of parameters corresponding to the second Gaussian distribution were added. Starting values were provided based upon apparent peaks in the histogram plots.

In all cases, the simpler model (single Gaussian) was chosen unless the more complex model (Dual Gaussian) was shown to be statistically superior. Comparison between models was assessed using a goodness-of-fit ANOVA. In addition, absolute goodness of fit (R^2^) was required to be > 95% for all accepted fits. Initial analyses were performed using data from the random repeat runs only, which yielded unimodal models in all cases. Mean RT values from the random runs were used to constrain the slow RT component for the subsequent analyses of RT data from the fixed-sequence blocks. Initial values for each model were provided based upon visual inspection of RT histograms. Analyses were conducted both by quarter to evaluate stability of RTs over the course of the training and collapsed across quarters to compare coefficients.

Comparison of %age fast responses across tDCS conditions was performed by comparing dual Gaussian models in which the ratio between fast and slow responses was assumed to be constant vs. those in which it was assumed to vary across conditions. The simpler model (all %ages equal) was accepted unless the more complex model (%ages different) was found to be statistically superior.

### tDCS

tDCS was applied by a saline-soaked pair of surface sponge pads (3 × 3 cm) using the battery-driven, NeuroConn DC-Stimulator MR (NeuroConn, Ilmenau, Germany). During the ERP section of the study, the participants received four stimulation conditions (Sham, Motor-cathodal, Visual-cathodal, Motor-anodal) using a constant current of 2-mA intensity applied for 30 minutes during the task performance (Fig. 1B). Each stimulation condition was administered on a separate day (at least 36 hours apart) for each participant in counterbalanced order. Finite-element modeling of electric field strength was performed on the MNI-152 head (6th generation, non-linear - T1-weighted), using the ROAST (69) toolbox in MATLAB. Electrical field strength outputted by ROAST as NIfTI volume was then mapped onto the standard averaged MNI surface (31) (Fig. 1C).

### EEG data acquisition

Continuous EEG along with digital timing pulses representing key presses was acquired through Brainvision Brainamp MR Plus amplifier system using 32 scalp active electrodes, impedances < 5 kΩ, referenced to the FCz electrode, bandpass filtered from 0.05 to 100 Hz, and digitized at 500 Hz. Data were re-referenced to average-reference and analyzed offline using BESA Research, version 6 (Brain Electric Source Analysis, BESA GmbH), EEGLAB(70), ERBLAB(71) and Matlab software, version 2017a (MathWorks).

Data were epoched from − 400 to + 200 ms relative to key motor response and were subjected to both automated (± 70 *μ*V at all scalp sites) and manual artifact rejection. Electrode positions that were removed to accommodate the tDCS pads were interpolated using Spherical Spline Interpolation (72). Epochs were subjected to time-frequency transformation using complex demodulation (73, 74) for frequencies of 4–50 Hz. Frequencies were sampled in 2-Hz steps; latencies were sampled in steps of 25 ms, yielding a time-frequency resolution of ± 2.83 Hz and ± 39.4 ms at each time-frequency bin (full width at half maximum).

As previously (31, 32), analyses focused on the − 200–0 ms pre-motor interval, relative to the prior 200 ms (−400 to −200 ms baseline). β-ERD values were calculated using temporal spectral evolution (TSE) defined as the relative power change at a time-frequency bin compared with the mean power over the baseline epoch for that frequency (43, 74).

#### Beamformer:

Intracranial sources of beta-activity were assessed using a Beamformer approach, as described previously (31, 32, 74, 75). β-ERD values were calculated using temporal spectral evolution (TSE) defined as the relative power change at a time-frequency bin compared with the mean power over the baseline epoch for that frequency (74, 76). Source modeling via Beamformer involves the following steps: 1) For each channel single-trial time-domain data are transformed into time-frequency domain in order to compute the complex time-frequency signal (74); 2) Complex cross-spectral density matrices is then computed for each trial. 3) A forward model is applied, and a lead-field matrix is estimated; and 4)

In computing the lead-field matrix we used the standardized finite element model (FEM) implemented in BESA. The FEM model provides a realistic approximation to the averaged head and uses three compartments: brain/CSF, skull and scalp to describes the electrical conductivity distribution inside of the head. The brain in Talairach space is then divided into a grid with a resolution of 5 mm^3^ and the beamformer image is constructed from values q(r) computed from every location on this grid. q values are then shown in % where q[%] = q*100.

This image is then extrapolated to a resolution of 1 mm^3^ and projected to an inflated brain image derived from an MRI of equal resolution. Since in the computation of beamformer image regional sources having three orthogonal vectors (i.e. radial, tangential and oblique) are used, projection onto an inflated brain surface more accurately represents the spread of the cortical activation. The overlap areas of the cortical projections obtained in HV and SZ groups are then used to determine the cortical regions with the highest q value which are then seeded with a virtual source (73, 77), revealing three distinct cortical regions at MNI coordinates: PMC/SMA [−26, −10, 74], Motor [−40, −22, 60], Visual [−48, −78, 2]. The goodness-of-fit (GOF) value of the model in controls is 90.0 SE ± 2.0 and in patients is 85.0 SE ± 2.0.

Coherence measures are then derived across this cortical network as a measure of functional connectivity (78–80), according to the formula:

$$C\prime_{xy}(f,t)=\frac{{\left|\sum_{n}S_{x,n}(f,t)\cdot S_{y,n}^{*}(f,t)\right|}^{2}}{\sum_{n}{\left|S_{x,n}(f,t)\right|}^{2}\cdot \sum_{n}{\left|S_{y,n}(f,t)\right|}^{2}}$$

Coherence ranges from 0 (no coherence) to 1 (maximum coherence). To determine the probability that coherence at a particular time-frequency sampling point is significantly higher than what is expected from random fluctuations is investigated based on an approach suggested by (81) and previously implemented and described by our group (74).

To investigate the probability that the coherence in sham differed significantly from the coherence in each of the other conditions, the individual subject mean coherence estimates were then subjected to a permutation cluster analysis (32, 82, 83). This approach is carried out in two general steps.

In the first step a Student’s paired t-test is carried out for every time-frequency (TF) bin to determine if there is a significant difference between the two conditions in the group. Here a cluster alpha level of 0.05 is set which allows us to serves as a test statistic for the next step of the analysis.

In the second step of the analysis, the clusters obtained in the preliminary parametric step are then submitted to permutation testing wherein the coherence data for sham gets systematically interchanged with the coherence data of the test condition. For each permutation, a new t-test is obtained per TF bin and a new test statistic (cluster-level summed t-values) is computed.

Here we have used 2000 permutations (drawn randomly without repetitions) from all possible permutations, i.e., 2^17^. From the distribution of the test statistics obtained from our permutations we then calculate the proportion of the test values that are larger than the value obtained from the initial cluster obtained in step 1. Hence if less than 5% of all values are larger than the initial test value it is assumed that the data of the two conditions are not interchangeable with a chance level greater than 95% i.e. (P < .05).

## Results

Initial analyses focused on between-group SRTT performance during the sham condition between Sz and HC, and its relationship to underlying neurophysiological (β-ERD) responses. Subsequent analyses focused on the magnitude and mechanism of tDCS effects across groups.

### Baseline performance

#### Mean RT analyses

In the random condition (Fig. 2A), there was a linear effect of Block (F_1,46_=18.5, p < .001) reflecting gradual improvement across groups. There was also a significant main effect of Group (F_1,46_=5.14, p = .03) reflecting longer RTs in the Sz group across blocks. As expected, the linear Block × Group effect was not significant (F_1,46_=1.00, p = .3). In the fixed condition (Fig. 2B), both the linear effect of Block (F_1,46_=85.4, p < .001) and the main effect of Group (F_1,46_=8.74, p = .005) were highly significant. In addition, there was a significant linear Group × Block interaction (F_1,46_=7.14, p = .01) reflecting a differential slope across groups.

In a combined analysis across the random and fixed condition, there was a significant main effect of group (F_1,46_=7.66, p = .008), a significant task × group interaction (F_1,46_=10.5, p = .002) reflecting the greater deficit observed in the fixed vs. random version of the task, and a highly significant 3-way linear task × block × group interaction (F_1,46_=23.8, p < 001), demonstrating that the differential change in slope across the two-tasks was statistically reliable across groups.

#### Comparison with traditional neurocognitive measures

**Figure 3:**
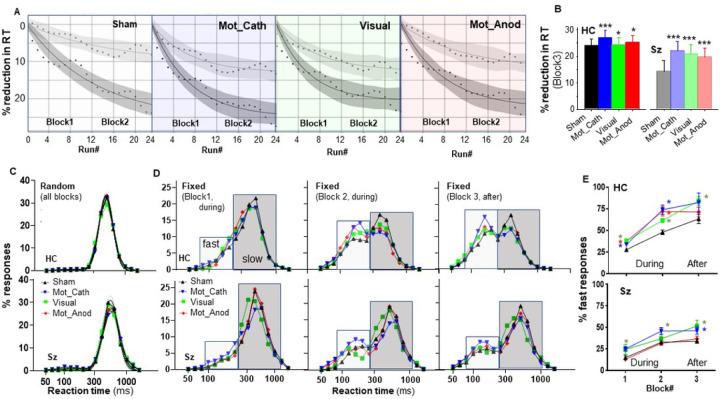
Effect of tDCS on SRTT behavior. *A*. Reduction in reaction time (RT) across runs for the Sham, Motor-Cathodal (Mot_Cath), Visual Cathodal, and Motor Anodal (Mot_Anod) condition. Runs 1–12 corresponding to block 1 and runs 13–24 corresponding to block 2. In all cases, curves fit well to an exponential improvement function. *B*. Effects of tDCS on mean RT across runs in block 3 by group. *C*. Superimposed RT histograms across blocks and stimulation condition in the Random task. Note unimodal response profiles. *D*. RT histograms for the fixed condition by Group, Block and Condition. Note bimodal RT distributions, with shift from slow to fast response mode in both groups. *E*. Percent fast responses by Group and Condition. * p<.05 across conditions for condition indicated by color vs. sham.

Sz was also associated with increased time to complete the Perdue Pegboard Task, along with reductions in neuropsychological performance across MCCB domains (Table 1). Relative increases in RT for the fixed vs. random version of the task correlated strongly with reduction in performance in the Assembly Trial of the Perdue Pegboard (r_*p*_ =.56, p < .001, Fig. 3C) as well as the Working Memory T-score of the MCCB (r_*p*_=.60, p < .001), with weaker correlations to Speed of Processing, Attention/Vigilance, and Visual Learning and Reasoning/Problem Solving (all p < .05). When these measures were entered into a simultaneous regression, the Purdue Pegboard (r_*p*_=.36, p = .033) and MCCB Working Memory (r_*p*_=.43, p = .01) were independently significant and accounted for 57.3% of the variance in SRTT performance (p = .009).

In Sz, performance on the SRTT did not correlate significantly with medication dose, illness duration, or symptom severity (all p > .2), although it did correlate with higher level measures including general function (GAF, r = .50, p = .028), function capacity (ILS, r = .50, p = .027) and participant (r=−.49, p = .027), but not parental (r = .18, p = .49), socioeconomic status.

#### Neurophysiology

In order to evaluate neurophysiological bases of the behavioral SRTT deficits in Sz, coherence analyses were performed on the pre-movement β-activity. As reported previously (31, 32), significant β-ERD was observed within the premotor, motor, and visual sensory regions, which mapped to the canonical dorsal attention, somatomotor and visual networks (84), respectively (Fig. 2E).

The magnitude of the β-ERD did not differ significantly between groups either on the surface or within any of the source regions (Table 2). In contrast, there was a highly significant Group × Connection interaction across connections (F_2,38_=5.29, p = .009), reflecting a significant reduction in coherence in the Motor-Visual pathway in the Sz versus HC group during the pre-movement period (F_1,39_=4.69, p = .037)(Fig. 2F). Across groups, the initial difference in RT in the fixed vs. random task correlated significantly with the baseline SMA-Motor cortex coherence (r_*p*_=.46, p = .003) (Fig. 2G).

#### Single trial analyses

In single-trial analyses, as in our previous study (52), data were best fit by a single Gaussian function during the random condition. Mean log-RT was significantly longer in the Sz (2.74 ± .004 log-ms; 550 ms) vs. HC (2.68 ± .002 log-ms; 479 ms) group (F_1,26_=220.5, p < .0001, Fig. 2G). For the fixed condition across blocks, data fit better to a 2-Gaussian model for both the HC (F_3,10_=152.1, p < .0001) and Sz (F_3,10_=70.0, p < .0001) groups, with separate populations of fast (“proactive”) and slow (“reactive”) responses (Fig. 2H). As with slow responses, the mean RT of the fast response mode was also ~ 50 ms longer in the SZ (2.44 ± .04 log-ms; 275.4 ms) than HC (2.36 ± 0.02 log-ms; 229.1 ms) group. In both groups, the percentage of fast responses increased progressively across blocks. Across all blocks, the percentage of fast responses was substantially lower for Sz than HC (F_1,24_=40.2 p < .0001) (Fig. 2I).

### Effects of tDCS

Effects of tDCS were assessed using both traditional (mean RT) and single-trial approaches. For the mean RT analyses, in order to control for the general psychomotor slowing in SZ, values from the fixed runs were normalized to those in the random runs and expressed as % reduction in RT relative to the mean random RT. As no effects of tDCS were observed for the random condition, a common normalization value was used across all conditions.

#### Mean RT

Mean RT was analyzed using an MMRM with factors of Group and tDCS Condition, and with Run as a covariate. During stimulation, there was a significant main effect of Group (F_1,46.2_=5.59, p = .022) as well as a highly significant Group × Run interaction (F_1,202684_=98.1, p < .001), reflecting reduced improvement over time in Sz versus HC participants. The main effect of Condition (F_3,202685_=90.2, p < .001) and the Condition × Group interaction (F_3,202685_=25.6, p < .001) were also strongly significant. Across groups, all tDCS conditions were significantly beneficial, with order Mot_Cath > Visual > Mot_Anod > Sham (Fig. 3A).

In order to evaluate the degree to which improvement was maintained following stimulation, a separate analysis was performed for block 3 (Fig. 3B). As for the earlier blocks, there were significant main effects for Group (F_1,46_=15.2, p < .001) and tDCS Condition (F_3,117847_=218.5, p < .001). For both groups, significant enhancement of plasticity was observed for all tDCS conditions Although the order of effectiveness was similar for the two groups, the relative degree of improvement was larger for the Sz than HC as shown by a significant Group × Condition interaction (F_3,117865_=46.3, p < .001).

#### Trial by trial analyses

In the trial-by-trial analyses, tDCS was again without effect on performance in the random condition in either group (Fig. 3C). In the fixed condition, bimodal fits were observed in all conditions, with the expected progressive shift from slow to fast responses across blocks (Fig. 3D). Consistent with the mean RT results, tDCS significantly increased the percentage of fast vs. slow responses for both HC and Sz across blocks, with largest effect for Motor-cathodal and Visual-cathodal stimulation (Fig. 3E, Table 2). Whereas Motor-anodal stimulation produced significant effects during stimulation in HC participants, no significant effects were observed in Sz. In Sz, both Motor-cathodal and Visual-cathodal stimulation produced effects that persisted following stimulation.

#### Neurophysiology

**Figure 4:**
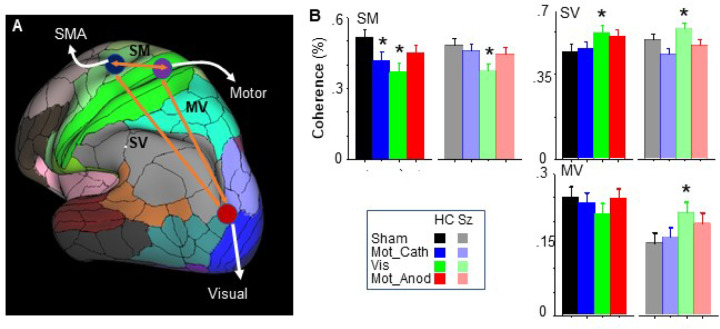
A. Schematic of interconnections analyzed using Beamformer coherence approaches. B-D: Cluster permutation analysis of coherence measures under indicated tDCS conditions for the SMA-Motor (SM), SMA-visual (SV) and Motor-Visual (MV) connections. *p<.05

tDCS effects on between-region coherence levels were assessed by univariate ANOVA with factors of tDCS Condition and Group (Fig. 4). tDCS significantly modulated coherence in both the SMA-Motor (F_3,114_=6.10, p < .001) and SMA-Visual (F_3,112_=4.08, p = .009) connections. In both cases, the modulation was most robust for stimulation over visual cortex. For Motor-Visual connectivity, there was a significant Group × Condition interaction across the Sham- and Visual-stimulation conditions, reflecting a non-significant reduction in coherence in the HC group vs. a significant increase in Sz (F_1,38_=7.00, p = .012).

In a multiple regression analysis of normalized RT vs. coherence across conditions controlling for group, RT showed oppositive changes in relationship to SMA-Visual vs. Motor-Visual cortex coherence, with RT decreasing with increasing SMA-Visual coherence, but increasing with increasing Motor-Visual cortex coherence, leading to a significant interactive effect of these coherences relative to RT across participants (F_1,103_=6.40, p = .013).

## Discussion

Sz is associated not only with persistent cognitive impairments, but also with impairments in cortical plasticity that limit the ability of individuals to improve performance with practice. tDCS enhances plasticity and learning capacity in healthy individuals (e.g. 11,13,29,57), but studies in Sz to date have yielded mixed results (e.g. 57,58,85). Here, we evaluated tDCS effects on implicit visuomotor learning using the SRTT combined with neurophysiological indices of interactions within a distributed premotor, motor and visual circuit.

Principal findings are three-fold. First, we confirm earlier reports of SRTT impairment in Sz (25), and demonstrate that the impairment as expected reflects a reduced shift from reactive to proactive responses. Second, we demonstrate that as in HC, tDCS applied over both motor and visual regions significantly enhances motor learning. Finally, we interrelate these with alterations in β-coherence between nodes of the underlying visuomotor circuit. Overall, these findings support a continued focus on development of tDCS for enhancement of plasticity-based interventions in Sz, as well as EEG biomarker-based approaches for evaluating underlying neural mechanisms.

### Visuomotor/procedural learning deficits in Sz

Cognitive dysfunction extends across a range of cognitive domains. However, over recent years there has been increasing realization that motor aspects of Sz are both important and understudied (6, 86). Nevertheless, optimal tests for the investigation of neural mechanisms underlying motor dysfunction need to be identified. Here, we benchmarked the SRTT against the Purdue Pegboard Test.

In Sz, deficits in manual dexterity measured using the Purdue Pegboard predate illness onset and are among the strongest predictors of conversion to Sz among prodromal individuals (87). In established Sz, reduced Purdue Pegboard performance is associated with diffuse white matter disorganization (88). Our study supports distributed network models of SRTT dysfunction in Sz, with particular emphasis on contributions of visuomotor connectivity (31). These findings are also consistent with prior studies demonstrating that impaired visual input into prefrontal cortex contributes to fragmented object recognition deficits in Sz (59, 89), which can also be disrupted in healthy individuals using TMS applied over the dorsal visual stream (60).

Deficits in SRTT performance are observed not only in Sz but also in Parkinson’s disease (PD), specific language impairment (SLI), and dyslexia (25). The shared deficit with PD suggests that SRTT dysfunction in Sz may be related to known dopaminergic contributions to motor function across disorders (8). However, the shared deficit with SLI and dyslexia argues that alternative mechanisms especially involvement of cortical sensorimotor networks (8) may also be critical and of specific relevance to Sz. For example, deficits in dorsal-stream visual performance in Sz contribute to low-level reading disturbances computationally similar to those observed in dyslexia and SLI (90–92).

Here, we observed two components of slowing in the SRTT. First, both proactive (fast) and reactive (slow) responses were ~ 50 ms slower across the random and fixed conditions. However, in the fixed condition, the majority of the deficit related to the reduction in the shift from slow to fast responses. How these patterns relate to those observed in other disorders remains to be determined. In our study, mean RT did not correlate with medication dose for either the random or fixed condition, arguing against medication-induced dopaminergic blockade as an underlying mechanism.

### tDCS

Our demonstration of tDCS effects on SRTT performance in healthy individuals using the traditional mean RT approach is consistent with extensive prior literature (e.g. 21,31,32). In addition, our single trial analysis confirmed that reductions in mean RT across runs correspond to motor learning, as reflected in a shift from slow to fast responses, rather than a change in mean RT of either response type independently.

In both mean RT and single-trial analyses, Motor-cathodal and Visual-cathodal stimulation proved most effective. Moreover, the degree of improvement in motor learning in Sz was significantly larger than in HC. Of note, although tDCS reduced the difference between the HC and Sz groups, it did not restore performance in Sz to control levels. In the present study, in order to remain compatible with prior literature, we used both group-mean field strength mapping and a low-density montage. Future studies with high-definition approaches (32), personalized mapping (93) and repeat administration may yield even further benefit.

### Neurophysiological outcome

As recently reviewed (94), EEG measures provide potential biomarkers of tDCS effect, but relatively few studies have been conducted to date. Both β-ERD (e.g. 43) and coherence measures among scalp electrodes were considered promising approaches. Here, we further refine the coherence approach by conducting the analyses within source-space using a Beamformer approach that we have previously validated relative to underlying fMRI connectivity patterns (31).

Here, we replicate the β-ERD source distribution in a new cohort of HC and Sz individuals, while also providing novel evidence for impaired Motor-Visual connectivity under non-stimulation conditions in Sz (Fig. 2F), and its potential amelioration especially by tDCS applied over visual cortex (Fig. 4B). Our finding of impaired β-coherence is consistent with a more general literature showing dysregulation of synchronous neural oscillations as a mechanism in pathophysiology of brain disorders (95) and in particular, abnormal β-frequency synchronous oscillations across cortical networks in Sz (96).

While β-coherence was significantly reduced in Sz and modulated by tDCS, the absolute magnitude of the β-ERD was not significantly affected. These findings are consistent with the increasing appreciation of Sz as a disorder of functional connectivity (e.g. 59,97,98,99). In contrast, the β-ERD has been shown to be reduced in primary motor disorders such as stroke, amyotrophic lateral sclerosis, dystonia and PD (100). Thus, as previously suggested source-localized EEG may provide an optimal approach for investigating network-level, as well as local effects of tDCS (20).

Thus, while disorders such as Sz and PD have shared SRTT deficits, underlying neural mechanisms may be significantly different. For example, a recent study did not find a significant effect on acute tDCS over motor cortex on either sequence learning or hemodynamic response in PD (101). The SRTT is well-suited to EEG-based analysis because of the large number of trials and the ability to “back-average” from the motor response. The present findings suggest that simultaneous EEG recording, especially when combined with network-level analysis, may assist in differentiating underlying neural mechanisms across disorders.

### Limitations

Although we show significant correlated effects of tDCS on behavior and network coherence, several limitations of the study should be considered. First, we used a low-density, non-personalized montage. Especially for the Visual cortex stimulation, current flow was not optimized to the region of greatest network-level impairment. In a more recent study in HC, we found that high-definition tDCS over visual cortex led to significantly greater effect than with the present montage (32). Future studies using personalized, optimized high-definition montages are needed in both Sz and HC groups. Second, there are many other potential nodes of relevance to the SRTT, such as striatum and cerebellum (e.g. 35,53), that were not assessed. Finally, all Sz participants were receiving antipsychotic medication, which may have affected results although no correlations with dose were observed.

## Conclusions

In summary, motor learning is a fundamental skill to our daily lives. Motor performance and its dysfunction in Sz has been associated with poor social and functional outcomes and contribute to decreased quality of life, impaired work capacity, and a reduced life expectancy by 10–20 years (9), but underlying mechanisms and potential treatment approaches remain understudied. Here, we demonstrate that the SRTT combined with source-space EEG analysis may be used both as a method for investigating mechanisms of motor and procedural learning deficits in Sz, and as a mechanism to develop refined non-invasive brain stimulation approaches for modulation of ongoing functional connectivity impairments across relevant disorders.

## Figures and Tables

**Table 1:** Demographics

	Control		Schizophrenia		
	Mean	SD	Mean	SD	P-Value
**Age**	36	11	36	10	NS
**Gender (% female)**	8	38.1	6	23.1	NS
**Age**	34.7	10.7	35.0	10.0	NS
**Participant SES**	46.4	10.1	26.1	7.0	**<0.001**
**Parental SES**	44.1	14.0	39.8	10.9	NS
**Quick IQ**	102.8	7.3	97.5	9.5	0.053
**Purdue Pegboard (Trial 1)**	33.9	6.7	20.7	7.8	**<0.001**
**Purdue Pegboard (Trial 2)**	35.1	1.5	21.4	8.0	**<0.001**
**Purdue Pegboard (Trial 3)**	36.6	7.8	22.4	8.35	**<0.001**
**MCCB (SoP)**	48.7	8.6	34.8	11.7	**<0.001**
**MCCB (AttVig)**	49.7	8.9	36.9	11.5	**<0.001**
**MCCB (WM)**	49.2	8.3	38.1	13.0	**0.002**
**MCCB (VerL)**	45.4	8.0	36.7	8.4	**0.001**
**MCCB (VisL)**	40.4	12.3	33.4	15.8	**0.120**
**MCCB (RPS)**	50.0	12.4	38.8	9.1	**0.002**
**PANSS Positive**	-	-	13.1	3.5	-
**PANSS Negative**	-	-	15.3	4.9	-
**PANSS Cognitive**	-	-	10.9	3.4	-
**Medication dose (CPZequiv)**	-	-	976.3	864.1	-
**Illness duration (yrs)**	-	-	13.1	7.6	-
**GAF**			49.5	12.8	

SES = Socioeconomic status (Edinburgh scale). MCCB = MATRICS Consensus Cognitive Battery. SoP = Speed of processing; Attvig = Attention/Vigilance; WM = Working memory; VerL = Verbal Learning; VisL = Visual Learning; RPS = Reasoning and Problem Solving; PANSS = Positive and Negative Symptom Scale; CPZe = chlorpromazine equivalents; GAF = General Assessment of Function.

**Table 2:** Comparison across blocks and condition by group

Ctl	Overall		Sham v. Mot_Cath		Sham v Visual		Sham v Mot_Anod	
	F (1,48)	p	F (1,24)	P	F (1,24)	P	F (1,24)	p
**Block1**	*6.37*	*0.001*	*7.17*	*0.013*	*28.3*	*<.0001*	*11.8*	*0.0022*
**Block 2**	*11.4*	*<.0001*	*23.6*	<.*0001*	*17*	*0.0004*	*20.68*	*0.0001*
**Block3**	*54.6*	*<.0001*	1.51	0.23	*5.76*	*0.025*	1.07	0.31
Sz	Overall		Sham v. Mot_Cath		Sham v Visual		Sham v Mot Anod	
	F	P	F	p	F	p	F	P
**Block1**	*3.411*	*0.025*	*7.36*	*0.012*	2.4	0.13	0.46	0.51
**Block 2**	*2.943*	*0.042*	*9.43*	*0.005*	0.55	0.47	0.13	0.73
**Block3**	*3.95*	*0.014*	*7.01*	*0.014*	*7.21*	*0.013*	0.51	0.45
